# Characterisation of the Subaquatic Groundwater Discharge That Maintains the Permanent Stratification within Lake Kivu; East Africa

**DOI:** 10.1371/journal.pone.0121217

**Published:** 2015-03-23

**Authors:** Kelly Ann Ross, Elisée Gashugi, Augustin Gafasi, Alfred Wüest, Martin Schmid

**Affiliations:** 1 Department of Surface Waters – Research and Management, Eawag: Swiss Federal Institute of Aquatic Science and Technology, Kastanienbaum, Switzerland; 2 Institute of Biogeochemistry and Pollutant Dynamics, ETH: Swiss Federal Institute of Technology, Zurich, Switzerland; 3 Department of Chemistry, UR-CST: University of Rwanda, College of Science and Technology, Kigali, Rwanda; 4 Laboratory of Physics of Aquatic Systems, EPFL: École Polytechnique Fédérale de Lausanne, Lausanne, Switzerland; University of Vigo, SPAIN

## Abstract

Warm and cold subaquatic groundwater discharge into Lake Kivu forms the large-scale density gradients presently observed in the lake. This structure is pertinent to maintaining the stratification that locks the high volume of gases in the deepwater. Our research presents the first characterisation of these inflows. Temperature and conductivity profiling was conducted from January 2010 to March 2013 to map the locations of groundwater discharge. Water samples were obtained within the lake at the locations of the greatest temperature anomalies observed from the background lake-profile. The isotopic and chemical signatures of the groundwater were applied to assess how these inflows contribute to the overall stratification. It is inferred that Lake Kivu’s deepwater has not been completely recharged by the groundwater inflows since its turnover that is speculated to have occurred within the last ~1000 yrs. Given a recent salinity increase in the lake constrained to within months of seismic activity measured beneath the basin, it is plausible that increased hydrothermal-groundwater inflows into the deep basin are correlated with episodic geologic events. These results invalidate the simple two-component end-member mixing regime that has been postulated up to now, and indicate the importance of monitoring this potentially explosive lake.

## Introduction

Lake Kivu formed its current structure following its ∼ 400 m lake-level rise and initiation of hydrothermal activity ∼ 10 ka [1, 2]. The lake consists of a permanently stratified anoxic monimolimnion that reaches 486 m depth, and a seasonally stratified mixolimnion within the top 65 m. Several sources of subaquatic groundwater discharge (SGD) into the northern part of the Main Basin of Lake Kivu are responsible for maintaining this form. A disruption of this stratification from a mixing event could result in the expulsion of a detrimental amount of CO_2_ and CH_4_ that have been accumulating within the monimolimnion. These gases have been accruing for over hundreds of years with the current partial pressure of CH_4_ equivalent to 60 km^3^ at standard temperature and pressure, and CO_2_ equivalent to 300 km^3^ at standard temperature and pressure. The last lake-mixing event is hypothesized to have occurred as recently as ∼ 750-1000 years ago [3]. Currently, the possibility of a lake-turnover, consequent of gas oversaturation within the monimolimnion, is particularly limited by the steep density gradient observed at ∼ 260 m depth and the hydrostatic pressure in the deepwater [4].

The persistence of this strong density stratification in the monimolimnion requires (1) sustained hydrothermal inputs (warm SGD) that are maintained at specific depths by freshwater inputs (cold SGD), and (2) a dilute mixolimnion maintained by cold SGD and surface inflows [5]. Three major density gradients (pycnoclines) are observed below the pycnocline that separates the mixolimnion from the monimolimnion. Increasingly warm SGD has been lifting the lower boundary of these pycnoclines since the onset of measurements in 1974 [6]. A recent warming of the deepwater of the lake has been observed, which is probably attributed to increased temperature or discharge of the SGD [7]. The warm groundwater consists of salt and CO_2_ that sustains the density stratification, whereas CH_4_ produced by microbial activity in the anoxic layer weakens the stability of this stratification [8]. A similar stratification scheme is observed for the separate basin Kabuno Bay. However, the deepwater of this basin has a higher conductivity and dissolved CO_2_ concentration relative to even the deepest waters in the Main Basin of Lake Kivu [9, 10].

The sources of SGD into the monimolimnion account for ∼ 20% of the total inflows [5]. The absolute discharge of these sources was estimated via a 1D advective-diffusive model applied to reproduce the CH_4_ profile and the salinity gradients that define the major pycnoclines [8]. The model required steady-state conditions within the density stratified layers of the lake, and is therefore overparameterised to a certain extent. Upward advection as a result of SGD yields an estimated residence time of 800-1000 years for the gases within the monimolimnion [8]. In addition to advection, double-diffusive convection in Lake Kivu modifies the gas, salinity, and temperature structure produced by these sources. Double-diffusion works to level out the steep density gradients, and removes heat from the deepwater faster than the salts and gases [11]. The differences in diffusion between heat, and gas/salt further stabilise the stratification in the deepwater, thereby lowering the possibility of degassing from the increased hydrothermal inflows [11, 12].

Although SGD plays a vital role in maintaining the structure of Lake Kivu, the subsurface hydrology in the Kivu Basin has not been studied. The water table geometry of the groundwater system is probably very complex, as is observed in lake-groundwater studies in other tectonically active regions [13–16]. Major fractures to the north of Lake Kivu align from the summit crater of Nyiragongo into the Main Basin, where similarly lineated faults are observed in the bathymetric map and high resolution seismic profiles of the sublacustrine setting [2]. The inhomogeneity of the geologic-surrounding makes classical hydrogeology work using piezometers not applicable in the Lake Kivu Basin. However, understanding the geochemical properties of groundwater coupled to the lake is a crucial factor in determining the future structure of the lake.

The aim of the present study was to determine the locations and chemical properties of SGD in Lake Kivu as a base for assessing the relevance of the different SGD sources for driving physical and biogeochemical processes in the lake. Our investigations were based on CTD-profiles and chemical/isotope analyses conducted on groundwater/lake water from within the lake, and assuming for the interpretation a horizontal homogeneity of the lake water [5]. The application of geochemical tracers assisted in determining the groundwater-lake water system dynamics and validate the presence of SGD. The isotopic ratios helped to decipher different water masses in addition to determining the subsurface environment containing the groundwater, and where the groundwater is recharging. Eight sources of SGD were located using conductivity and temperature profiling over the course of two years. The aquatic chemistry, including the *δ* ^18^O and *δ* ^2^H isotopic values of the groundwater, were used to characterise the discharge of these sources. A complex mixing-regime is hypothesized to exist within Lake Kivu, which reacts to episodic geologic events that increase the heat, salt, and CO_2_ content of the deepwater. This has implications for the present and future socio-economics of the region; including the possibility of a lake overturn event and the current extraction of methane from the deepwater for power production.

## Regional and hydrological setting

**Fig 1:**
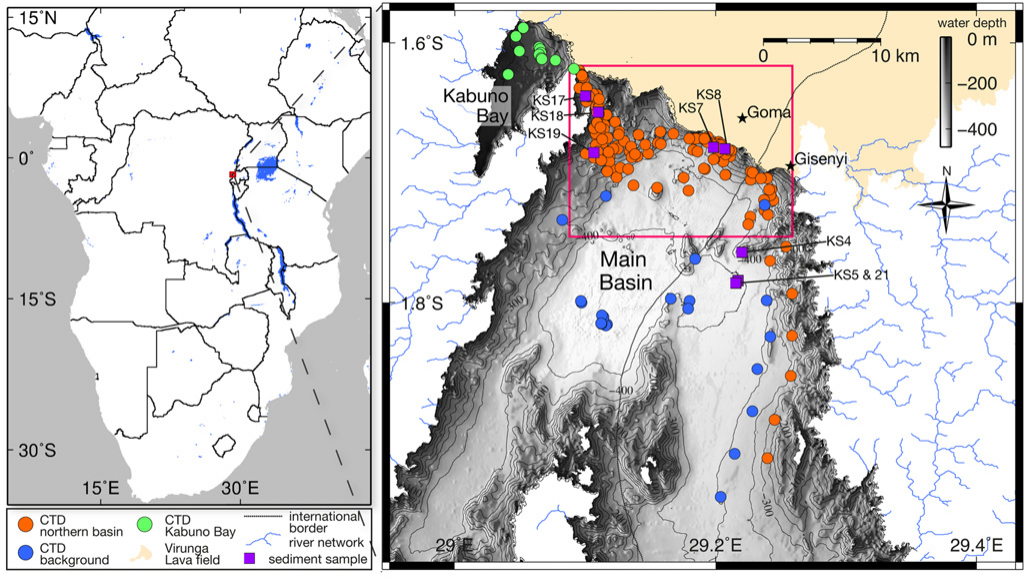
A total of 290 profiles were taken in the Main Basin (orange and blue), and 11 profiles in Kabuno Bay (green). The background profiles (blue) were used to calculate an average profile for determining the temperature differences at the locations of SGD. A total of 7 sediment samples were analyzed to determine the geochemical differences at the locations of cold (KS7, 8) and warm (KS17, 18, 19) SGD relative to the background (KS4, 5, and 21). The pink outlined box represents the location analyzed for temperature differences of SGD in Fig. 3. The Virunga Lava field north of the basin, which is void of surface flows, could represent an area of high aquifer recharge. (Inset: Location of the Main Basin in Lake Kivu; Albertine Rift Valley in eastern central Africa)

Lake Kivu is situated at an elevation of 1463 masl. It has approximately a 1:2 ratio of lake-water (2385 km^2^) to watershed (4940 km^2^) area, and a lake volume of 549 km^3^. There have been no rivers in the relatively flat, 685 km^2^, area to north of the lake (‘Virunga lava field’ in Fig. 1) observed within at least the last decade. Recent hydroclimate data suggests drought-like conditions within the last few decades in this region [17] that is also recorded in the Kivu Basin, albeit with considerable interannual fluctuations [18]. Subaerial and subaquatic volcanic activity in the Lake Kivu Basin has defined the current structure of the lake, and the presence of subaquatic volcanic features has been outlined by previous research [2].

Lake Kivu is recharged predominately by direct precipitation (1404 mm/yr), followed by input of more than 100 streams and rivers [19]. The outflow of the lake is maintained by the Ruzizi River discharge that drains into Lake Tanganyika, and by evaporation [19]. The total hydrological output of 6.3 km^3^/yr; 2.7 Ruzizi + 3.6 evaporation, and the inputs (1.6 rivers + 1.3 SGD + 3.5 precipitation) are given by Muvundja et al. [18]. The overall uncertainty in the water budget is approximately 15 to 20% and the seasonal and annual variability in the lake’s volume is within this range of 1 km^3^/yr [18]. The 1.3 km^3^/yr input by SGD calculated by Schmid et al. [8] is ∼ 40% of the Ruzizi outflow. This value is relatively well constrained by the salt budget, as the SGD is the major source of salt to the lake. The calculated inputs at 180 and 250 m depth are one order of magnitude greater than those below these depths and contribute up to 90% of the total SGD into the lake. The SGD drives an average upwelling rate ranging between ∼ 0.15 m/yr at the main pycnocline and ∼ 0.7 m/yr in the upper monimolimnion. This upwelling is the main driver for the transport of nutrients [20] and dissolved gases [21, 22] from the deepwater to the mixolimnion. With regard to subsurface drainage, so far no work has been conducted in the Kivu watershed.

## Materials and Methods

### CTD profiling and temperature mapping

A total of 301 CTD profiles were collected from January 2010 to March 2013 using a RBR XR-620 probe (Fig. 1). Locations and sampling dates are given in the supplementary S1 Table. No special permissions for field work on Lake Kivu were required, because the study was part of a joint scientific project involving the University of Rwanda (former Kigali Institute of Science and Technology) and the Institut Supérieur Pédagogique de Bukavu (DR Congo). Initial sampling was conducted at different depths along the northern shore of the lake, based on previous observations of temperature peaks in CTD profiles [23] and temperature microstructure profiles [11] taken in this region. Further profiling was done in the central part of the Main Basin as a background reference, and southward in the eastern part of the lake to confirm the initial assumption that no important SGD can be observed in this region far from the main submerged volcanic structures.

Subsequently, profiles were sampled more densely around locations where significant signals from SGD were found in the initial campaign. Furthermore, a spatial hydrological analysis was calculated using the ARC-GIS tools *Flow Direction* and *Flow Accumulation*, on the bathymetric map produced in a previous study [2]. The resulting stream network was used to trace dense groundwater that was suspected to be flowing along the basin floor via CTD profiling. A subset of initially 12 and subsequently 16 profiles was selected to be sampled every few months in order to evaluate the temporal variability of the signals from the SGD [24]. Most CTD profiles covered the full vertical extent from the water surface to the local bottom depth.

Temperature signals caused by the SGD were detected in the profiles using an algorithm implemented in MATLAB version R-2013a. Each of the profiles were linearly interpolated using a 0.5 m median-average window. A background profile was averaged using a median filter applied to 25 profiles taken in the deep basin (Fig. 1). The temperatures and conductivities, corrected to a temperature of 25 °C, of all profiles and the background profile are given in the supplementary S2 and S3 Tables, respectively. Within each depth range, the maximum difference in temperature was extracted, where the differences were positive for warm sources of SGD, and negative for cold sources of SGD. The depth of maximum difference was then used to extract the temperature and conductivity of the profile at this depth. The conductivity was not used to locate SGD because of spikes produced in the profile when the probe entered the sediment. These spikes are not filtered from the interpolating due to dragging along the sediment floor during profiling. The resulting temperatures were plotted for analysis using the free software GMT 4.5.8, and the functions described within Wessel and Smith (1991) [25]. On occasion the small temperature variability observed in is not a direct consequence of the inflows, but a result of the algorithm used for determining the temperature differences in combination with a slight difference in the depth of the gradient at different locations. It is suspected that these minor depth differences are an effect of internal waves observed within the lake [26].

### Chemical and isotopic analysis

Sampling for chemical analysis was conducted over the course of three field campaigns in January 2010, October 2010, and January 2012. The results of the chemical analysis of all individual water samples are given in the supplementary S4 Table. Water samples were collected with 5-liter Niskin bottles. The exact depths of the water samples at the sources of SGD were determined by consulting the RBR probe data prior to analysis, which was attached to the bottom of the Niskin bottle. For the areas of warm groundwater discharge, and for depths below 200 m, the open valve at the top of the Niskin bottle was capped with a balloon to prevent sample loss from vigorous outgassing upon ascent. Samples were filtered immediately with a 0.4 micron Whatman™ disc filter for analysis of major cations and anions. Sample portions were acidified with ultra pure HNO_3_ for metal analysis, and portions were also oxidized with ultra pure H_2_O_2_ and shaken in order to convert H_2_S for total sulphide (∑S) measurements. The latter samples were measured for SO_4_^2−^ in the lab. Major ions were measured at Eawag in Switzerland by ICP-MS (Perkin-Elmer ELAN 5000), and ICP-OES (SPECTRO-CIROS), and on a Metrohm ion chromatograph (733 IC Separator Center, 732 IC Detector, 762 IC Interface). Averages were taken from multiple methods of analysis for most of the major cations and anions. The *δ* ^18^O and *δ* ^2^H isotopic values were determined using a Picarro L1102-i laser analyzer, and the *δ* values are reported in ‰ relative to the standard SMOW. Chemical analysis of poor quality and major outliers were discarded prior to any interpretation of the data. Alkalinity was titrated in the field with the use of a 716 DMS Titrino™ (Metrohm) and 0.1 mol/L HCl. Vertical profiles for pH were recorded *in situ* in October 2010 for the lake background profile, and at the locations of groundwater inflows, with a Seabird SBE-19 conductivity, temperature and depth (CTD) probe. The pH was calibrated onboard the research vessel using a 3-point calibration curve that spanned the lake-water pH values. The CO_2_ was calculated from the pH and alkalinity using the free software PHREEQC provided by the USGS. Furthermore, the consistent negative charge balance, which was suspected to be a result of MgCO_3_ precipitation prior to the analysis in the lab, was offset by calculating Mg^2+^ using PHREEQC. The calculated values result in no difference to the interpretation of the data. Water density was calculated as a function of temperature, salinity, CO_2_, and CH_4_ for the background samples, using the Chen-Millero equation [27]. Here within, salinity is defined as the total of dissolved solids and gases in the water mass.

The geochemical analysis for TOC, HCO_3_^−^, and S of the sediment samples is given by Bhattarai et al. [28]. An additional analysis was conducted here on sample KS4 in order to determine the Fe concentration using an Axios, PANalytical wave-length dispersive X-ray fluorescence spectrometer (WD-XRF). The resulting Fe concentration was then compared to the XRF counts within the top 2 cm of the core KV-04 (taken at the same time as KS4), reported in a previous study [3]. A transfer function was then applied to the counts in the top 2 cm of core KV-21 (KS21), in order to calculate the concentration of Fe here. A different XRF scanner was used for all other sediment samples, and therefore the transfer function was not applicable.

## Results

### Background profile

**Fig 2:**
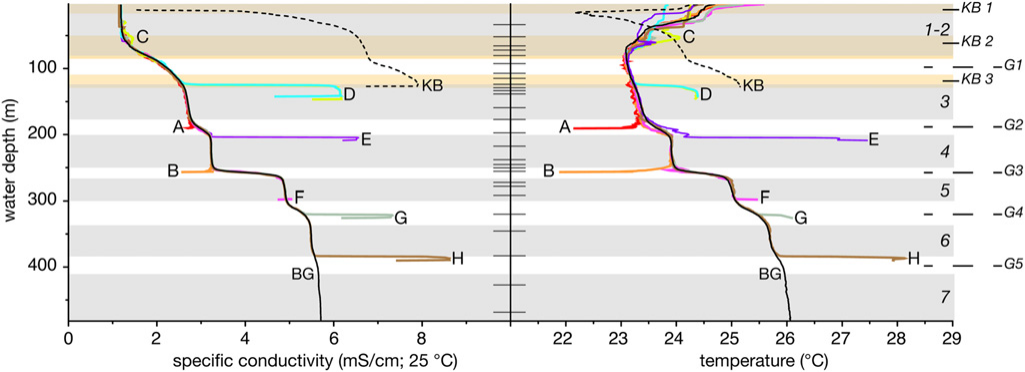
The conductivity and temperature profiles observed at the location with the maximum temperature difference for each source in Fig. 3 (A-H). The black line is the average background profile (BG). The hatching at the centre represents the depths where water samples were taken in the Main Basin. The shading in the background marks the depth range where the chemical and isotopic values for the samples were averaged; grey shading for the Main Basin, and beige shading for Kabuno Bay (only one sample was analyzed for each of the Kabuno Bay layers). The codes given on the righthand side are the layers analyzed for the parameters listed in Table 1, 2, and 3. ‘G-’ are the gradients referred to the in main text.

CTD profiling of the background locations in Fig. 1 reveals the first pycnocline that separates the mixolimnion from the monimolimnion from 65 m up to 150 m depth (G1), and the major pycnoclines in the deepwater centred at ∼ 190 (G2), 260 (G3), and 315 (G4) m water depth, with a minor pycnocline centred at ∼ 385 (G5) m depth (Fig. 2). A background profile is also delineated for Kabuno Bay (‘KB’ in Fig. 2), which is located to the northwest of the Main Basin (Fig. 1) and separated by a saddle at ∼ 11 m depth. The Kabuno Bay profile is thermally stratified to ∼ 11 m depth at which point the salinity and temperature increase dramatically to 140 m, with two main pycnoclines centred at ∼ 15 and 120 m depth.

In agreement with previous observations [5], the background profiles sampled sufficiently far from the influence of SGD showed high horizontal homogeneity and little temporal variability. The standard deviation of temperature below 80 m depth in the 25 profiles forming the background profile generally ranged between 0.01 and 0.02 °C and reached a maximum of 0.06 °C at 80 m depth. The latter was probably largely induced by small vertical changes of the main gradient layer (G3), as a temperature difference of 0.06 °C corresponds to a vertical displacement of less than 0.5 m at this depth. Similarly, for specific conductivity the standard deviations were typically around 0.005 mS/cm outside the gradient layers, and between 0.01-0.02 mS/cm inside the gradient layers, with a maximum of 0.08 mS/cm observed at 250 m depth corresponding to a vertical displacement of less than 0.5 m.

### Spatial distribution of SGD

#### Cold SGD

**Fig 3:**
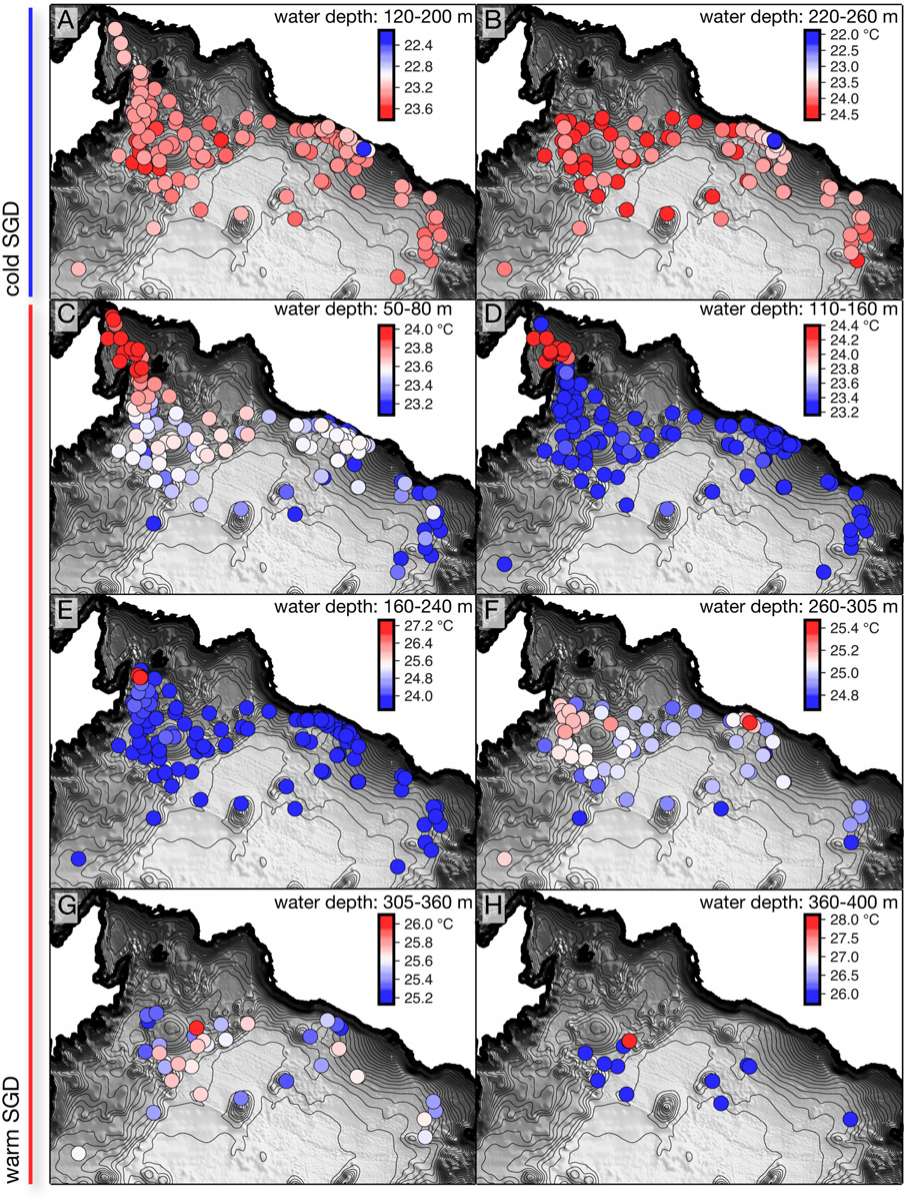
The 290 profiles, represented by the circles, were analyzed to locate the maximum difference of temperature from the background profile at the depths specified. The temperatures at the point of maximum difference is visually represented by the colour scale in the upper right hand corner of each image. The scale is increasing for cold SGD, and decreasing for warm SGD. The profile points are plotted with the coldest temperature on top for A-B, and warmest temperatures on top for C-H. The high variability observed in the temperatures in some locations, particularly for source B, is due to the temporal and spatial changes in the depth of the gradient (G3).

Two point sources of cold SGD were located in the northeastern part of the Main Basin, directly west of the submerged flank of the phreatomagmatic cone Mount Goma. The location of sources A and B in Fig. 3 are represented by the coldest temperatures observed within the respective frames. Point source A in Fig. 2, at ∼ 190 m depth, deviates from the background temperature profile by 1.52°C and conductivity profile by 0.26 mS/cm. The next three coldest temperatures at the same location and depth as A are on average 0.8°C warmer than the observed point source. Directly to the west of A, point source B is observed in Fig. 3B at a depth just above the main density gradient (G3 in Fig. 2). This source is 2.41°C cooler and has a 1.33 mS/cm lower conductivity than the background profile. Most profiles within 1-2 km of this location present cooler temperatures than the background profile at a similar depth. Furthermore, the coldest temperatures at this depth are typically observed to the southeast of this source, and particularly along the eastern flank of Mount Goma. In addition to the strong signals from the point sources A and B, small negative temperature peaks were observed in numerous profiles in this region in the depth range between G1 and G2 (e.g. negative offset in profiles A, B, and F in Fig. 2). An additional source of cold SGD was expected at the top of the lower pycnocline, G4. Although the point source was not located, a profile ∼ 500 m southeast of source A and B yields a maximum temperature difference that is 0.4°C cooler than the background profile. The sources of cold SGD all appear to be located along a possible fracture where Mount Goma abuts the steep sloping wall to the north [2].

#### Warm SGD

A total of six sources of warm SGD (C-H in Fig. 2) are located almost entirely in the northwestern part of the basin at the volcanic platform delineated by previous research [2]. Source C appears as a warm plume in the conductivity and temperature profile at ∼ 50 m depth in Fig. 2. A cluster of profiles revealing this plume are located at the channel that separates Kabuno Bay and the Main Basin by a saddle at ∼ 11 m water depth in Fig. 3C. The warmest signal is 0.74°C warmer and 0.21 mS/cm saltier than the background profile. This plume is observed up to 10 km away from the channel. Since the plume enters the lake in the mixolimnion, where the background profile temperature varies seasonally due to meteorological forcing, the signal of the plume may be influenced by the temperature variation of the mixolimnion water entrained into the plume. The calculated differences in the plume versus the background profile may therefore be affected by seasonal changes unrelated to the effective plume signal. Source D fills a small basin up to 120 m water depth, which is located southwest of the channel that connects Kabuno Bay to the Main Basin in Fig. 3D. There is a sharp temperature and salinity gradient (profile D in Fig. 2) that separates the water below this depth from that above it. The water below the gradient is 1.08°C warmer, and 1.98 mS/cm saltier than the background profile. There are two profiles taken directly outside the basin to the south that present similar temperatures, however, additional profiling in the area did not yield the observation of a warm plume from this source. Point source E was observed along the slope of the shoreline to the southeast of source D in Fig. 3 E. This source is located at the bottom of the pycnocline G2 and has a temperature that is 3.56°C warmer, and is 2.99 mS/cm saltier than the background profile (profile E in Fig. 2). However, no warm plume was observed in the profiles within the vicinity of this point source. Directly south of this source, there is a warm plume centred at ∼ 285 m depth that is observed in a cluster of profiles bordering a magmatic dome feature in Fig. 3F, which is described by Ross et al. [2]. This plume is a maximum of 0.14°C warmer and only 0.02 mS/cm saltier than the background profile. Additionally, a point source just below the depth of this plume (profile F in Fig. 2) was observed directly west of the cold source B (Fig. 3F), which is 0.42°C warmer, and 0.12 mS/cm saltier than the background profile. The profiles between these two locations are all colder than either the plume or point source itself (Fig. 3F). Two other point sources are observed in a ∼ 1 km wide channel just southeast of the same dome feature in Fig. 3G and H, directly below G4 and G5 in Fig. 2. These sources are 0.58 and 2.3°C warmer, and 0.78 and 3.03 mS/cm saltier, respectively, than the background profile. Although the implied plumes from the point sources of warm SGD are difficult to trace beyond their location of discharge, many of the profiles taken within the deep basin (> 400 m water depth) express small peaks in temperature of up to 0.1°C at the bottom of the pycnoclines G3, G4, and G5.

### Aquatic chemistry

The aquatic chemistry of the background profile is presented within layers 1-7 in Tables 1, 2, and 3, which represent the mixed layers of the lake above and below its pycncolines (G1-5 in Fig. 2). A similar representation is given for Kabuno Bay, where 3 layers are presented (KB1-3 in Fig. 2).

**Table 1:** Aquatic chemistry: state variables for density calculations.

	**depth^a^**	**alkalinity**		**pH**		**CO_2_**	**conductivity**		**salinity**		**temperature**		**density**
location	m	mEq/L	±		±	mmol/L	mS/cm	±	‰	±	°C	±	g/cm^3^
**background**													
1	0-65	13.30	*0.14*	8.74	*0.11*	0.04	1.211	*0.030*	1.106	*0.029*	23.54	*0.32*	0.9982
2	65-75	16.18	*1.27*	7.79	*0.43*	0.50	1.446	*0.088*	1.337	*0.087*	23.10	*0.02*	0.9985
3	130-180	31.80	*0.96*	6.64	*0.03*	13.17	2.683	*0.064*	2.603	*0.068*	23.33	*0.08*	0.9995
4	200-250	36.98	*0.47*	6.41	*0.02*	25.45	3.225	*0.062*	3.185	*0.068*	23.91	*0.07*	0.9999
5	270-300	59.84	*1.24*	6.14	*0.00*	71.45	4.880	*0.027*	5.062	*0.032*	24.98	*0.04*	1.0014
6	330-370	71.70		6.07	*0.01*	96.94	5.430	*0.085*	5.721	*0.102*	25.59	*0.13*	1.0020
7	> 400	73.70	*0.00*	6.05	*0.00*	103.40	5.675	*0.030*	6.019	*0.037*	26.01	*0.05*	1.0022
**Kabuno Bay**													
1	10	19.70		7.45		1.30	1.631		1.520		23.60		0.9985
2	50	93.50		6.33		67.43	6.517		7.071		23.88		1.0035
3	122	117.10		6.33		80.25	7.888		8.869		25.13		1.0046
**cold SGD**													
A	135	29.48		6.65		12.22	2.624		2.540		23.20		0.9995
B^b^	253	33.45		6.37	*0.02*	25.96	3.190	*0.012*	3.147	*0.013*	23.39	*0.19*	1.0002
**warm SGD**													
C	47	14.60		7.80		0.44	1.405		1.295		23.93		0.9983
D	134	83.78	*2.59*	6.14	*0.00*	95.82	6.143	*0.001*	6.599	*0.001*	24.36	*0.00*	1.0033
E	205	70.15		5.82		170.00	6.543		7.104		27.06		1.0039
F	287	59.56		6.12		74.34	4.919		5.109		25.05		1.0018
G	–	–		–		–	7.314		8.104		26.06		–
H	387	123.40		–		107.9^c^	8.629		9.885		27.90		1.0049

^a^ average based on hatching in Fig. 2

^b^ average based on two samples from 2012. A third sample from 258 m depth taken in 2010 was not included in the averaging, see supplementary S4 Table

^c^ The CO_2_ was estimated using the [HCO_3_^−^]:[CO_2_] ratio from source D

– indicates that the sample was not analyzed, or was discarded, for this parameter

± is the standard deviation calculated from layers or sources where more then one sample was analyzed

**Table 2:** Aquatic chemistry: major cations and anions.

	**depth^a^**	**Cl**		**Si**		**Ca**		**Na**		**K**		**Mg^b^**
location	m	mmol/L	±	mmol/L	±	mmol/L	±	mmol/L	±	mmol/L	±	mmol/L
**background**												
1	0-65	0.78	*0.01*	0.14	*0.01*	0.13	*0.03*	4.62	*0.06*	2.09	*0.02*	3.72
2	65-75	0.84	*0.00*	0.20	*0.02*	0.33	*0.01*	4.89	*0.10*	2.22	*0.04*	4.78
3	130-180	1.53	*0.19*	0.62	*0.02*	1.72	*0.04*	9.11	*0.38*	3.86	*0.06*	8.45
4	200-250	1.70	*0.09*	0.83	*0.04*	2.04	*0.13*	10.86	*0.28*	4.51	*0.12*	9.50
5	270-300	2.32	*0.05*	1.25	*0.02*	2.89	*0.33*	16.61	*0.24*	6.67	*0.16*	16.54
6	330-370	2.78	*0.09*	1.34		3.16	*0.00*	18.55	*0.09*	7.36	*0.76*	21.12
7	> 400	3.89	*1.81*	–		3.33	*0.04*	20.03	*0.33*	7.35	*0.02*	21.77
**Kabuno Bay**												
1	10	0.81		–		0.73		4.81		2.21		6.25
2	50	2.07		–		1.97		16.72		7.69		33.13
3	122	2.27		–		1.62		21.065		9.06		42.78
**cold SGD**												
A	135	1.50		0.67		1.37		9.42		3.69		7.72
B^c^	253	1.48	*0.01*	0.75	*0.00*	1.72	*0.16*	8.85	*0.89*	3.33	*0.36*	9.48
**warm SGD**												
C	47	0.74				0.43		4.64		2.03		3.91
D	134	2.22	*0.33*	1.85	*0.32*	2.46	*0.28*	21.32	*0.56*	8.13	*0.55*	25.52
E	205	11.51		0.88		2.54		46.34		5.84		12.20
F	287	2.67		1.30		2.71		16.83		6.74		16.61
G	–	–		–		–		–		–		
H	387	2.83		2.82		2.95		35.34		10.82		37.01

^a^ average based on hatching in Fig. 2

^b^ calculated to offset the charge balance error as explained in the methods section

^c^ average based on two samples from 2012. A third sample from 258 m depth taken in 2010 was not included in the averaging, see supplementary S4 Table

– indicates that the sample was not analyzed, or was discarded, for this parameter

± is the standard deviation calculated from layers or sources where more then one sample was analyzed

**Table 3:** Aquatic chemistry: redox sensitive elements and stable isotopes.

	**depth^a^**	**∑S**		**Fe**		**Mn**		***δ*^18^O**		***δ*^2^H**	
location	m	mmol/L	±	mmol/L	±	mmol/L	±	‰	±	‰	±
**background**											
1	0-65	0.16	*0.00*	0.000	*0.000*	0.000	*0.000*	3.9	*0.1*	29.9	*0.1*
2	65-75	0.16	*0.01*	0.000	*0.000*	0.000	*0.000*	4.1	*0.1*	29.5	*0.3*
3	130-180	0.17	*0.01*	0.000	*0.000*	0.006	*0.000*	3.3	*0.1*	26.1	*0.6*
4	200-250	0.18	*0.03*	0.000	*0.000*	0.005	*0.000*	2.6	*0.1*	22.4	*1.0*
5	270-300	0.19	*0.03*	0.001	*0.001*	0.005	*0.000*	1.1	*0.1*	15.4	*0.5*
6	330-370	0.32	*0.07*	0.001		0.006		0.4	*0.1*	12.5	*1.2*
7	> 400	0.27	*0.01*	–		–		0.2	*0.0*	10.6	*0.1*
**Kabuno Bay**											
1	10	0.23		0.000		0.000		2.0		17.0	
2	50	0.37		0.477		–		-2.7		-4.1	
3	122	0.27		0.218		–		-2.8		-4.7	
**cold SGD**											
A	135	0.16		0.001		0.006		3.2		25.8	
B^b^	253	0.21	*0.03*	0.015^c^		–		1.9	*0.1*	18.1	*1.2*
**warm SGD**											
C	47	0.11		0.000		–		3.2		22.7	
D	134	0.33	*0.02*	0.289	*0.009*	0.006		-2.3	*0.1*	-2.5	*0.5*
E	205	0.32		0.005		–		0.7		13.4	
F	287	0.20		0.001		0.006		1.3		17.4	
G	–	–		–		–		–		–	
H	387	0.31		0.071		–		-2.7		-2.8	

^a^ average based on hatching in Fig. 2

^b^ average based on two samples from 2012. A third sample from 258 m depth taken in 2010 was not included in the averaging, see supplementary S4 Table

^c^ measured in the sample from 258 m depth from 2010, see supplementary S4 Table

^−^ indicates that the sample was not analyzed, or was discarded, for this parameter

^±^ is the standard deviation calculated from layers or sources where more then one sample was analyzed

The density is calculated from the state variables in Table 1 and compared to the density calculated for the sampled sources of SGD A-H. From this, we can determine in which layer we expect the measured sources of groundwater to stratify if disregarding plume formations. The cold sources A and B have densities of 0.9995 and 1.0002 g/cm^3^, respectively. We would therefore expect source A to stratify within the mixed layer 3 and source B to stratify at the bottom of layer 4. In regards to the warm sources of SGD, we expect that source C with a density of 0.9983 g/cm^3^ would stratify at the bottom of layer 2. The sampled warm source F will stratify between layer 5 and 6 according to its density of 1.0018 g/cm^3^, however, this warm source was sampled at the location of its plume near the magmatic dome. The sampled sources D, E, and H should all stratify at the bottom of the lake according to their densities, which are greater than the density of 1.0022 g/cm^3^ determined in layer 7. Additionally, theses sources are within the density range observed in layers KB2 and KB3 of Kabuno Bay (Table 1).

**Fig 4:**
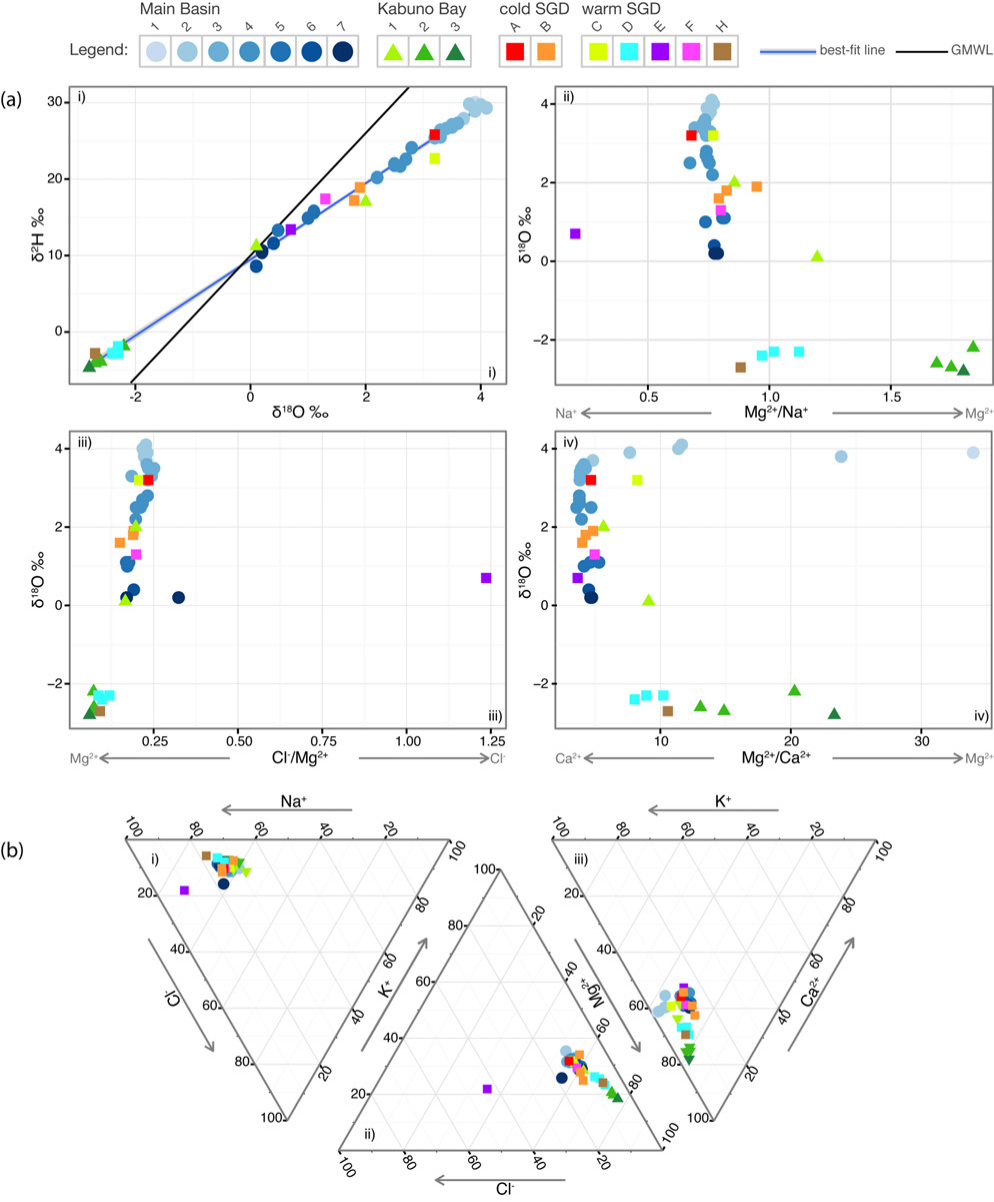
Geochemical plots for a complete characterisation of the SGD into Lake Kivu. (a) The *δ* ^18^O v. *δ* ^2^H values represent the end-member mixing within Lake Kivu and are compared to the Global Meteoric Water Line (GMWL). ii) Plot of Mg^2+^/Na^+^ v. *δ* ^18^O, ii) Cl^−^/Mg^2+^ v. *δ* ^18^O, and iv) Mg^2+^/Ca^2+^ v. *δ* ^18^O. The gray arrows below each graphic indicate increasing concentrations of the specific elements. (b) Ternary plots of i) Cl^−^-K^+^-Na^+^, and ii) K^+^-Mg^2+^-Cl^−^ and iii) Mg^2+^-Ca^2+^-K^+^. The percentage of each element represented by the sample is given by the axis that increases in the direction of the arrow. A legend is indicated at the top and describes the grouping illustrated in Fig. 2 and listed in Table 1.

The *δ* ^18^O and *δ* ^2^H isotopic values in Lake Kivu were determined in order to delineate groundwater-lake water mixing. The *δ* ^18^O versus *δ* ^2^H isotopic values yields a mixing line that crosses the global meteoric water line (GMWL) in Fig. 4ai. The warm SGD sources D and H plot above the GMWL along with the samples measured below the main gradient in Kabuno Bay. The deep samples from Kabuno Bay (KB2 and 3) and the warm sources D and H all have *δ* ^18^O values < -2 and *δ* ^2^H values < 0. The warm sources C, E, and F plot with the background water samples and cold sources of SGD A and B below the GMWL, and trend toward an increasingly evaporative signal with *δ* ^18^O values > 0 ‰ and *δ* ^2^H values > 10 ‰. The cold source B plots at the same position as the sample KB1 taken in the epilimnion of Kabuno Bay. The shallowest background sample plots the furthest from the line, and the two lines cross at the deepest background sample and a sample taken within the main gradient of Kabuno Bay. A similar profile for the background and Kabuno Bay samples were measured recently by Katsev et al. [7].

Cations and anions measured in the background samples 1-7 increase with depth in the lake at a relatively constant proportionality for Mg, K, Na, and Cl. Furthermore, Ca concentrations within the mixolimnion decreased by a factor of ∼ 6 relative to the decrease in these elements (Table 2), as a consequence of its control by aragonite precipitation. Similar observations were also recorded by Pasche et al. [20], and Degens et al. [29], and a single mixing line was inferred. A two component end-member phenomenon was originally observed for the background profile, and samples taken from tributaries and terrestrial hydrothermal springs by Degens et al. [29]. The authors concluded that a single mixing line is a consequence of mixing two water masses; one being the evaporated-surface lake water system enriched in *δ* ^18^O (3.5 ‰), and the other the meteoric-hydrothermal reservoir that is *δ* ^18^O depleted (-3 ‰). In this study, we observe notable exceptions from a constant proportionality of cations and anions when the sources of SGD and the samples from Kabuno Bay are compared to the background samples from the Main Basin.

The deep samples from Kabuno Bay (KB2 and 3) are enriched in Mg:Na (Fig. 4aii) compared to all other samples, and greatly enriched in Mg:Ca (Fig. 4aiv) relative to the samples and sources taken within the monimolimnion of the Main Basin. Furthermore, the warm sources D and H are more enriched in Mg:Na and Mg:Ca, with respect to the same samples. Both the warm sources D and H and the deep Kabuno Bay samples KB2 and 3 are less enriched in Cl:Mg relative to all other samples and sources in Fig. 4aiii, where the most enriched sample is the warm source E followed by a sample taken within layer 7 of the background. Another significant observation is that Mg:Na and Mg:Ca increase with sampling depth in Kabuno Bay (Fig. 4aii and 4aiv). Furthermore, the warm source H has a lower Mg:Na ratio but a higher Mg:Ca ratio than the warm source D. The warm source F, sampled at its plume location, plots with the background samples at the same depth. In regards to the cold sources, A has a similar *δ* ^18^O, Mg:Na (Fig. 4aii), and Cl:Mg (Fig. 4aiii) relative to the warm source C. One sample of source B is relatively enriched in Mg:Na and slightly less enriched in Cl:Mg, similar to the deeper sources (Fig. 4aii and 4aiii).

From the ternary plots in Fig. 4b, we observe that all the samples and sources cluster closely together for the Cl-K-Na plot in Fig. 4bi, with the highest relative Na and Cl and lowest K values observed in the warm source E. More trending is observed in Fig. 4bii and 4biii that is similar to that described for Fig. 4a, where the deep Kabuno Bay samples cluster separately from the background samples and sources, but more similarly to the deep sources D and H. Furthermore, as the proportion of Mg decreases, there is a concomitant increase in K, and Ca trending towards the background samples and sources in the monimolimnion (Fig. 4bii). The effect of the relatively high proportion of Cl in source E is observed in Fig. 4bii, but absent when replacing the Cl with Ca in in Fig. 4biii.

The redox sensitive elements S, Fe, and Mn are presented in Table 3. The concentration of S remains constant in layers 1-5 at ∼ 0.17 mmol/L and then increases to ∼ 0.29 mmol/L below G4. Pasche et al. [20] measured a relatively constant concentration of S^2−^ below 160 m depth (∼ 0.23 mmol/L), concluding that the precipitation of pyrite is responsible for Fe-depletion from the water column. We observe a molar ratio of Fe:S in the background sediment samples KS4 and KS21 (Table 4) of ∼ 1:2. This ratio implies the presence of FeS_2_ in the sediment, which was also inferred from the downcore measurements of S and Fe conducted in a previous study [3]. If we assume that all S is present as FeS_2_ in the sediment, then we can roughly estimate an increase in S and Fe in the sediment by a factor of ∼ 5 and 6 at the locations of the cold and warm SGD in the Main Basin, respectively. A concentration of 0.001 mmol/L of Fe was measured in the water column below the main density gradient, whereas 1-2 orders of magnitude higher concentrations are observed in the warm sources (D and H in Table 3), and in one sample from the cold source B. Conversely, Mn concentrations below the mixolimnion remain constant throughout the measured background samples and within the sources of SGD at ∼ 0.006 mmol/L.

**Table 4:** Geochemistry of surface sediment samples.

	**depth**	TOC*	CO_3_^2−^*	S*	**Fe**
location	m	%wt	%wt	%wt	%wt
**background**					
KS4	181	6.48	48.00	1.33	1.26
KS5	451	7.02	23.00	1.31	–
KS21	450	7.70	20.00	1.65	0.96
**cold SGD**					
KS8	157	8.86	5.00	6.74	–
KS7	296	7.78	25.00	6.69	–
**warm SGD**					
KS17	145	5.04	36.00	7.15	–
KS18	207	8.43	6.00	10.94	–
KS19	317	9.75	17.00	6.83	–

* Parameters taken from Bhattarai et al. [28]

## Discussion

### Cold SGD

Cold SGD appears to flow from a fracture along the northern shoreline of the lake. Two sources were identified at this location (A and B) that emerge as point sources. SGD similar to the characteristics of A and B might also be discharging along the eastern border of Mount Goma, where additional point sources of cold SGD were observed. The large variability observed in the profiles at the location of source A up to the depth of the mixolimnion could imply additional sources of groundwater here, including diffusive flow. Source A appears similar in its isotopic and chemical composition to source C that is located at the channel connecting Kabuno Bay (Fig. 3), which suggests a similar end-member. It is apparent from the chemical analysis in Table 3 that source B has a lower *δ* ^18^O isotopic value (1.9 ‰) than the water sampled above it in layer 4 (2.6 ‰), and a higher value than that sampled below in layer 5 (1.1 ‰). From the combination of its cooler temperature, its density, and the lower cation and anion content relative to that of the background *δ* ^18^O, we infer that this source is stratifying at its depth of discharge and diluting the warm and salty water that is pushed upwards by advection from below. Since the water above this source has a higher *δ* ^18^O isotopic value instead of an expected value between the range of 1.1-1.9 ‰, this would imply that source B is one component in the mixing regime and the lake water a second component.

The measured salinities of 2.54 ‰ for source A, and 3.15 ‰ for source B from the samples taken at the location of SGD (Table 1) are lower and higher, respectively, than the salinities of 2.71 and 2.46 ‰ estimated from the conductivity profiles in Fig. 2. This could imply that our samples are contaminated with lake water. Schmid et al. [8] calculated discharge rates of 0.69 and 0.47 km^3^/yr, and salinities of 2.1 and 2.7 ‰ at the same depth as these two sources, respectively. From the estimated salinities given here, we propose that the actual discharge for source A is higher, and for source B is lower, in order to produce the salinities currently observed in the lake under steady-state conditions. This would affect the rate of upward advection estimated in the lake at these depths.

The S content in source B (0.21 mmol/L, Table 3) is nearly equivalent to that found above and below this source in layers 4 and 5. Furthermore, Fe was detected at concentrations 10*x* higher in one sample at the location of source B (0.015 mmol/L), than that found below G3 (0.001 mmol/L, Table 3). This implies that Fe manifests from the groundwater discharge at source B. We have no information on the oxidation state of S within the SGD. However, it is well possible that it is discharged as SO_4_^2−^ from the phreatic aquifer likely supplying the cold source here. Further evidence of pyrite precipitation at the location of the cold SGD is contained within the sediment, which has 5*x* higher pyrite at the depths just below sources A and B (Table 4).

### Warm SGD

The six sources of warm SGD were located predominately at the volcanic platform to the northwest [2]. Volcanic gas/fluid-groundwater interactions have often been cited as one of the major factors controlling hydrothermal discharge, and are suspected to be the primary reason for the onset of the density stratification in Lake Kivu [2, 30, 31]. From the enrichment of cations and alkalinity, it is clear that the sampled warm sources C, D, E, F, and H, and samples KB2 and KB3 all originate from hydrothermal sources. Mineral leaching by CO_2_-rich geothermal induced waters increases the composition of alkalinity and cations in the system, while maintaining an equilibrium with the *p*CO_2_ [32].

Based on the densities calculated for sources E, F, G, and H, we assume that these sources all contribute to SGD into the deepwater of the Main Basin (Table 1). Furthermore, we assume that source D is filling the basin observed in Fig. 3, but is likely not overflowing into the Main Basin. Therefore, we have located four sources of warm SGD equivalent to that suggested by Schmid et al. [8] to be discharging below the main pycnocline (G3). However, the high salinities and temperatures of the sources located in our study (Fig. 2 and Table 1) would suggest a significantly lower discharge of these sources than the cumulative discharge of 0.15 km^3^/yr calculated by Schmid et al. (2005) [8] that contained lower salinities and temperatures.

Source C is measured at 50 m depth directly at the channel that connects the Main Basin to Kabuno Bay, where a dominant plume is observed here (Fig. 3C). The warm and relatively salty water of this source presents a similar chemistry to that found in the background profile in layer 3 and in source A (Fig. 4a), which could indicate geothermally warmed groundwater that initially feeds source A. The density of this source indicates stratification within layer 2, however, the dominant plume observed at this location implies that it mixes within the mixolimnion in layer 1. We infer that this source has little effect on the salinity of the mixolimnion compared to river inflows, upwelling from the monimolimnion, evaporation and outflow via the Ruzizi River.

The high proportion of Mg relative to Na, Ca, and K in samples KB2 and KB3 and next in sources D and H, relative to the background samples, indicates that Mg is readily being leached in the hydrothermal system. Source E has the lowest Mg:Na and highest Cl:Mg content suggesting a different hydrothermal source here. These results are coherent with the ratio of [HCO_3_^−^]:[CO_2_] that determines the pH of the system, which is the same for samples KB2 and KB3 (1.2), slightly lower for source D (0.9), and 3*x* lower for source E (0.4) (Table 1). This suggests a higher geothermal *p*CO_2_ at source E exists compared to that of the system that feeds into Kabuno Bay and sources D and H. Overall, the similarities in the chemistry and isotopic composition of KB2 and KB3, and that of sources D and H, are remarkable (Table 1, 2, and 3). We therefore suggest that the same hydrothermal source contributes to the SGD in Kabuno Bay and the Main Basin, where there is likely a hydrogeologic connection between the two. The decreased Mg:Na, Mg:Ca, and Mg:K (data not shown) in sources D and H relative to samples KB2 and KB3 (Fig. 4aii and 4aiv), could simply be from hydrothermal percolation through the different lacustrine sediment in the Main Basin that is often aragonite rich [3, 33]. The encompassing hydrothermal system has a *δ* ^18^O isotopic composition of ∼ -2.7 ‰, where the slightly higher isotopic composition determined in source D (-2.3 ‰) is likely due to dilution by lake water within the basin here. Comparatively, the hydrothermal system of source E has a significantly higher *δ* ^18^O isotopic value (0.7 ‰).

The concentration of S in the sources D, E and H, and Kabuno Bay samples KB2 and KB3, is the same as that found in the background samples in layers 6-7 (Table 3). We assume that S is precipitated immediately as FeS_2_ upon discharge, and that the remaining Fe measured forms a complexation with HCO_3_^−^. With regard to the spatial distribution of the S in the sediment at the location of the warm sources, the highest S and lowest CO_3_^2−^ concentrations are given at KS18 near source E, which could support the theory that more FeS_2_ is precipitated from the sources with a lower HCO_3_^−^ concentration (Table 1 and b).

### Groundwater and lake-water mixing dynamics

Conventional hydrochemical techniques are not always easy to apply in a rift environment where subsurface hydrothermal systems commonly exist in proximity to active volcanoes [13–14, 34]. Although it is not possible to quantify the rate of SGD into the Main Basin from locating and characterizing the sources alone, we are able to postulate how the sources of SGD contribute to the stratification in the Main Basin based on certain principles. Firstly, groundwater flow systems can be schematized into two major vertical zones: a shallow zone of active, fast water flow and a deeper zone of relatively slower flow and longer residence times. Secondly, the dominance of upward vertical advection, the horizontal homogeneity within the background profiles, and the lack of turbulent diffusion in Lake Kivu allows us to estimate hydrothermal discharge based on a simple box-model.

**Fig 5:**
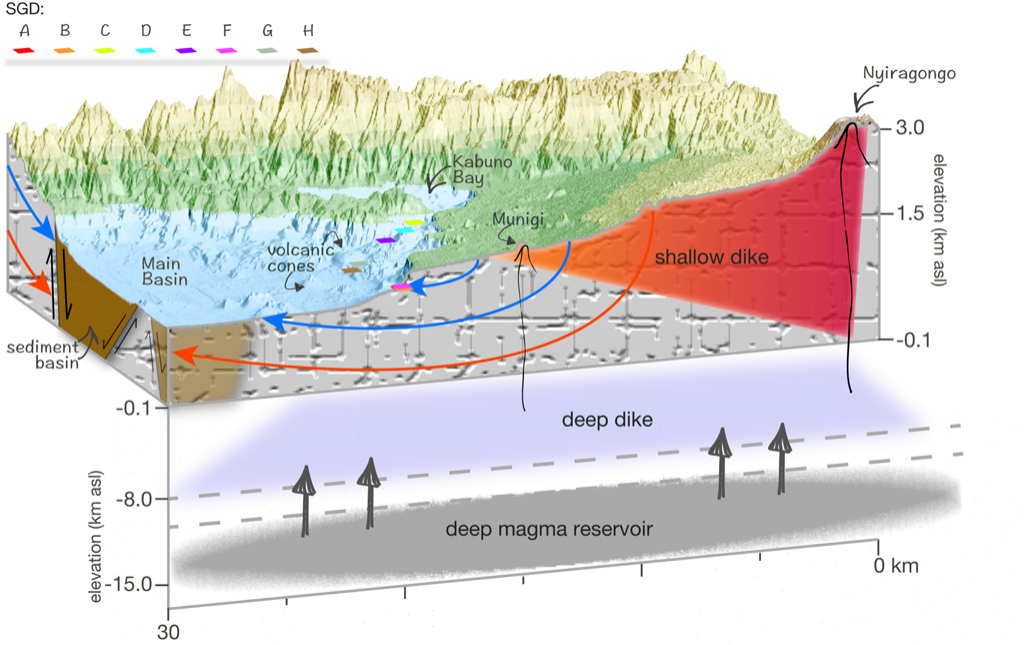
Conceptual model of the interaction of the SGD with the volcanic/geothermal system present beneath Lake Kivu and north of the lake towards Nyiragongo. The illustrated deep magma reservoir, deep dike, and shallow dike, including depths and extent, were modelled after Wauthier et al. [36]. The warm SGD C, D, E, G, and H trend along a N-W direction towards Kabuno Bay. The cold SGD A and B, and warm source F are located just west of the phreatomagmatic cone Mount Goma [2]. The blue and red arrows depict the convective groundwater flow paths within the fractured system. The blue arrows indicate shallow groundwater and the red arrows indicate deeper groundwater, which interacts with the geothermal system.

To begin, we hypothesize that within the Lake Kivu Basin there is a zone containing a major unconfined aquifer, with a deeper underlying, volcanic fractured aquifer existing in the rift floor (Fig. 5). Within the Lake Kivu catchment, the Virunga lava field (Fig. 1) is devoid of surface flows, and precipitation in this area probably contributes significantly to aquifer recharge [18]. Furthermore, faults connecting this aquifer to the Main Basin have a northeast-southwest alignment with the Nyiragongo volcano chain [2]. In general, the hydrologic properties of fault zones are thought to be highly anisotropic. Vertical faults can serve as conduits for horizontal flow along the fault, or barriers to horizontal flow across the fault, or both. Additionally, faults are conduits for vertical flow and can connect aquifers over different depths [35]. A study conducted in Yellowstone revealed individual thermal systems aligned at depth to form a large, contiguous geothermal reservoir [14]. In Lake Kivu, the cold sources of SGD are probably vertically isolated from the geothermal source (Fig. 5), and therefore their temperatures should represent the average ambient temperatures in the region. However, it is possible that the cold SGD along the northern shoreline of Lake Kivu is vertically and horizontally connected to the hydrothermal system. This is indicated by the proximity of the point source F to that of A and B, the one sample of source B that appears similar to that of the warm sources D and H, and the similarity of the warm source C with that of A. Furthermore, the hydrothermal source F may be connected to the plume observed at a similar depth at the volcanic platform. All of which would imply that the hydrothermal and cold groundwater systems are vertically and horizontally connected. The warm sources D and H, and the sources that contribute to the deepwater profile in Kabuno Bay, appear to be discharged from a deep contiguous geothermal reservoir that is trending northwest, and likely includes source G based on its location (Fig. 5 G).

Mixing within a regional groundwater flow system has the effect of averaging the isotopic composition of various groundwaters from different recharge environments. The isotopic composition of groundwater in such cases converges on the mean weighted value of all recharge contributions. If the recharge of regional groundwater occurs at higher altitudes where the average temperatures are lower, precipitation will be isotopically depleted compared to the local aquifers recharged at lower elevations. ^18^O/^16^O ratios are further fractionated through rock-water interactions in the aquifer, where the rate of equilibrium reactions will proceed faster in geothermally heated waters [37]. The *δ* ^18^O isotopic values of KB2 and KB3 are the same as those of source H, and therefore preclude isotopic changes from rock-water interactions. The warm source E has a higher *δ* ^18^O isotopic value of 0.7 ‰ that is attributed to a different hydrothermal source, which is likely recharged at significantly lower elevations than H. This is also assumed for the cold source B with a *δ* ^18^O isotopic value of 1.9 ‰. We suggest that the cold sources A and B, and the warm sources C and E are discharging from a local unconfined aquifer. However, source E is connected to a geothermal reservoir that enriches the NaCl and *p*CO_2_ in this source. Furthermore, sources D and H contributing to the lake, and KB2 and KB3 below the main gradient in Kabuno Bay, are probably associated with local deep thermal sources that are isolated from the unconfined aquifer, such as that depicted in Fig. 5.

The contribution of these sources to the stratification of the lake appears to go beyond a simple linear-mixing system. Here we postulate at least a four-component end-member mixing regime: (1) the lake profile following a lake mixing event, (2) SGD sources A, B and C, (3) SGD sources G and H, and (4) SGD source E. It is apparent from the *δ* ^18^O versus *δ* ^2^H isotopic values (Fig. 4ai) that the background profile is trending towards an evaporative source in the seasonally stratified mixolimnion, and a negative source in the deepwater recharged by SGD. The dominance of upward advection in the lake [8], and the low self-diffusion coefficient of water [38], preclude significant downward fluxes by turbulent diffusive transport under present conditions. Given the estimated discharge of the SGD [5], the isotopic composition of the deepwater in the lake should have approached that of the SGD since the likely onset of stratification with the lake-level rise some 10 ka BP. A coherent explanation for these contrasting observations is that a mixing event has been effectively transporting evaporated surface water to the deepwater in the recent past. This could also explain why the cold SGD B has isotopically depleted *δ* ^18^O values relative to the layers above this source. If the lake were to mix under the present conditions, the resulting profile would have a *δ* ^18^O value of 2.8 ‰. The contribution of the SGD would then begin to produce the large gradients observed in the lake. We propose that both sources H and E are contributing to the lake stratification, with a higher contribution from source H than E (Fig. 4aii, iii, and iv). Specifically, the layers 5 and 6 appear to receive a higher contribution from source H, while layer 7 receives a higher contribution from source E (Fig. 4).

There has been an ∼ 1% increase in conductivity below 320 m depth observed in CTD profiles taken in the deepwater (data not shown), which is constrained to have occurred between May 2007 and July 2008. Additionally, a higher Cl concentration and alkalinity is measured in the lake below 320 m in this study relative to that conducted by Pasche et al. [20] in 2007. Given the *M*_*w*_ 5.9 earthquake beneath the lake on February, 2008 [39], it is possible that the conductivity increase is attributed to this event. Lake Albano has recently increased in CO_2_ as an effect of seismic events in the area [40], and a comparative study in Yugama Lake observed an increase in Cl^−^ and SO_4_^2−^ concentrations in correlation with volcanic tremors. In the latter system, several hot springs and cold springs existed in different parts of the catchment, where the hot springs were aligned along major regional faults [34]. The complexity of this aquifer system may be similar in dynamics to that found at Lake Kivu, which is illustrated in Fig. 5. The increased Cl concentrations observed in Lake Kivu will have a greater effect on conductivity relative to increases in HCO_3_^−^ [41]. However, if the conductivity increase were a result of the sources G and H observed in this study below 320 m depth, then a discharge volume of 1.08 km^3^ from source G down to 370 m depth, and a volume of 0.24 km^3^ from source H below 370 m depth can be calculated. This cumulative SGD volume of 1.32 km^3^ is much larger than the volume of 0.12 km^3^/yr estimated by Schmid et al. [8]. Given that the last lake mixing event was expected to have occurred ∼ 750-1000 years ago [3], total SGD over this time period from episodic events would effectively produce the lake salinity-stratification observed today.

## Conclusions

Lake Kivu presents a dynamic system in which its stratification is highly influenced by groundwater inflows. The characterisation of these groundwaters is pertinent to predicting the future stratification of the lake. Net groundwater flow into lakes is typically calculated as the residual in the hydrologic budget, providing no information on the magnitude of inflow or outflow. Determining the location of these groundwater sources and identifying how their inflows contribute to the stratification in Lake Kivu is the first step to quantifying the SGD into the lake. Within the groundwater system in the Lake Kivu catchment, multiple locations of cold and warm SGD likely co-exist with varying heat and solute transport processes. The outcome of this research indicate that the lake conductivity and temperature profiles are a product of episodic geologic events within the lake. Such events implicate the importance of lake monitoring at Kivu. Additional studies should therefore be applied to confirm the temporal and spatial resolution of SGD into Lake Kivu. These results therefore serve as a substantial base for developing a transient model of the past and future density stratification in the lake.

## Supporting Information

S1 Table Metadata of the CTD (Conductivity, Temperature, and Depth) profiles used in the present study, including profile codes, and sampling times and locations. The profiles used for calculating the background profile are marked with (BG).
(TXT)

S2 Table Temperatures observed in the CTD profiles listed in S1 Table. The profiles were interpolated to 0.5 m intervals.
(TXT)

S3 Table Conductivities, corrected to 25 °C, observed in the CTD profiles listed in S1 Table. The profiles were interpolated to 0.5 m intervals.
(TXT)

S4 Table Results of the chemical analyses on water samples from Lake Kivu included in the present study. The sample codes correspond to the sampling locations in Table 3; BG: background; KB: Kabuno Bay.
(TXT)
